# Plastic roles of phenylalanine and tyrosine residues of TLS/FUS in complex formation with the G-quadruplexes of telomeric DNA and TERRA

**DOI:** 10.1038/s41598-018-21142-1

**Published:** 2018-02-12

**Authors:** Keiko Kondo, Tsukasa Mashima, Takanori Oyoshi, Ryota Yagi, Riki Kurokawa, Naohiro Kobayashi, Takashi Nagata, Masato Katahira

**Affiliations:** 1 Institute of Advanced Energy, Kyoto University, Gokasho, Uji, Kyoto, 611–0011 Japan; 2 Graduate School of Energy Science, Kyoto University, Gokasho, Uji, Kyoto, 611–0011 Japan; 3 Department of Chemistry, Graduate School of Science, Shizuoka University, 836 Ohya, Suruga, Shizuoka 422–8529 Japan; 4 Division of Gene Structure and Function, Research Center for Genomic Medicine, Saitama Medical University, 1397–1 Yamane, Hidaka-shi, Saitama 350–1241 Japan; 5 Institute for Protein Research, Osaka University, 3–2 Yamadaoka, Suita, Osaka 565–0871 Japan

## Abstract

The length of a telomere is regulated via elongation and shortening processes. Telomeric DNA and telomeric repeat-containing RNA (TERRA), which both contain G-rich repeated sequences, form G-quadruplex structures. Previously, translocated in liposarcoma (TLS) protein, also known as fused in sarcoma (FUS) protein, was found to form a ternary complex with the G-quadruplex structures of telomeric DNA and TERRA. We then showed that the third RGG motif of TLS, the RGG3 domain, is responsible for the complex formation. However, the structural basis for their binding remains obscure. Here, NMR-based binding assaying revealed the interactions in the binary and ternary complexes of RGG3 with telomeric DNA or/and TERRA. In the ternary complex, tyrosine bound exclusively to TERRA, while phenylalanine bound exclusively to telomeric DNA. Thus, tyrosine and phenylalanine each play a central role in the recognition of TERRA and telomeric DNA, respectively. Surprisingly in the binary complexes, RGG3 used both tyrosine and phenylalanine residues to bind to either TERRA or telomeric DNA. We propose that the plastic roles of tyrosine and phenylalanine are important for RGG3 to efficiently form the ternary complex, and thereby regulate the telomere shortening.

## Introduction

The end of the human chromosome, known as a telomere, has a repeated DNA sequence, d(TTAGGG)_n_^1^. Upon reduction of heterochromatin marks of a telomere, telomeric DNA is transcribed into telomeric repeat-containing RNA (TERRA), which has a repeated RNA sequence, r(UUAGGG)_n_^2,3^. These G-rich sequences form a G-quartet and fold into a G-quadruplex structure in the presence of monovalent cations such as K^+^ and Na^+^^4–6^. Although the functions of the G-quadruplex structure in a telomere are not fully understood, one of the important functions of a telomeric G-quadruplex is considered to be as a binding target for regulatory proteins^7^. Previously, it was reported that formation of the ternary complex between translocated in liposarcoma (TLS) protein, also known as fused in sarcoma (FUS) protein, and the G-quadruplex structures of telomeric DNA and TERRA leads to telomere shortening through repression of telomerase-independent telomere-elongation^8^.

**Figure 1:**
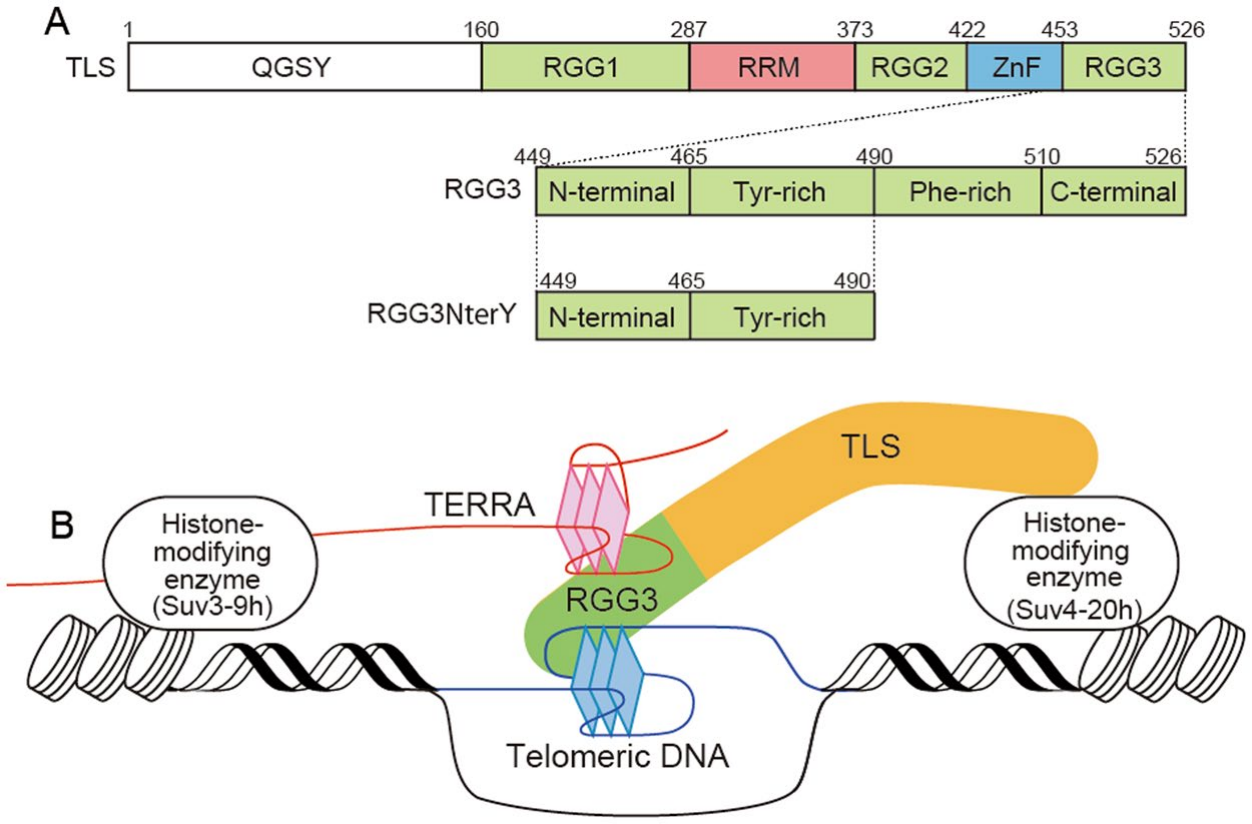
(**A**) Schematic illustration of TLS, RGG3, and RGG3NterY. (**B**) A proposed model^8^ describing the recruitment of histone-modifying enzymes by a ternary complex comprising TLS, telomeric DNA, and TERRA. The G-quartet planes of the G-quadruplex structures for telomeric DNA and TERRA are depicted.

TLS was originally found as a nucleic acid-binding oncogenic fusion protein^9^, which is located mostly in the nucleus and is responsible for numerous regulatory processes, including gene expression, mRNA/micro RNA processing, and maintenance of genomic integrity^10–12^. Then it was found that TLS functions in the regulatory processes not only through binding to RNA and DNA but also through binding to other proteins^13^. TLS comprises, from the N-terminus: a Gln-Gly-Ser-Tyr-rich (QGSY) region; the first Arg-Gly-Gly-rich (RGG) motif, RGG1; an RNA recognition motif (RRM); the second RGG motif, RGG2; a zinc-finger (ZnF) domain; and the third RGG motif, RGG3 (Fig. 1A). Only the RRM and ZnF domains are known to have structures, the remainder being considered as intrinsically disordered regions (IDR), which have no distinct structure.

As mentioned above, TLS interacts with the G-quadruplex structures of telomeric DNA and TERRA simultaneously. We previously showed that RGG3 of TLS is responsible for this ternary complex formation, by which histone methyltransferases Suv4–20 h and Suv3–9 h are recruited (Fig. 1B). These histone-modifying enzymes induce trimethylation of lysine 20 of H4 (H4K20) and lysine 9 of H3 (H3K9), which results in the promotion of heterochromatinization of the telomere. This leads to inhibition of telomerase-independent telomere-elongation, which is caused by homologous recombination of telomeric DNA, and consequently the telomere is shortened^8,13–16^. Therefore, the ternary complex comprising RGG3, telomeric DNA and TERRA plays a crucial role in the regulation of telomere length.

RGG3 comprises Tyr- and Phe-rich regions in the N- and C-terminal stretches. Importantly, phenylalanine and tyrosine residues are involved in the specific recognition of telomeric DNA and TERRA, respectively^17,18^. However, as there have only been biochemical studies on the interaction of RGG3 with G-quadruplex structures, the structural basis for the complex formation that triggers the telomere shortening is not fully understood. In the present study, we performed NMR analyses of an RGG3:telomeric DNA binary complex, an RGG3:TERRA binary complex, and an RGG3:telomeric DNA:TERRA ternary complex to obtain further insight into the interactions between RGG3 and G-quadruplex structures.

## Results and Discussion

### Telo24 and TERRA12 form a ternary complex with RGG3 like Telo22 and TERRA24 did

**Figure 2:**
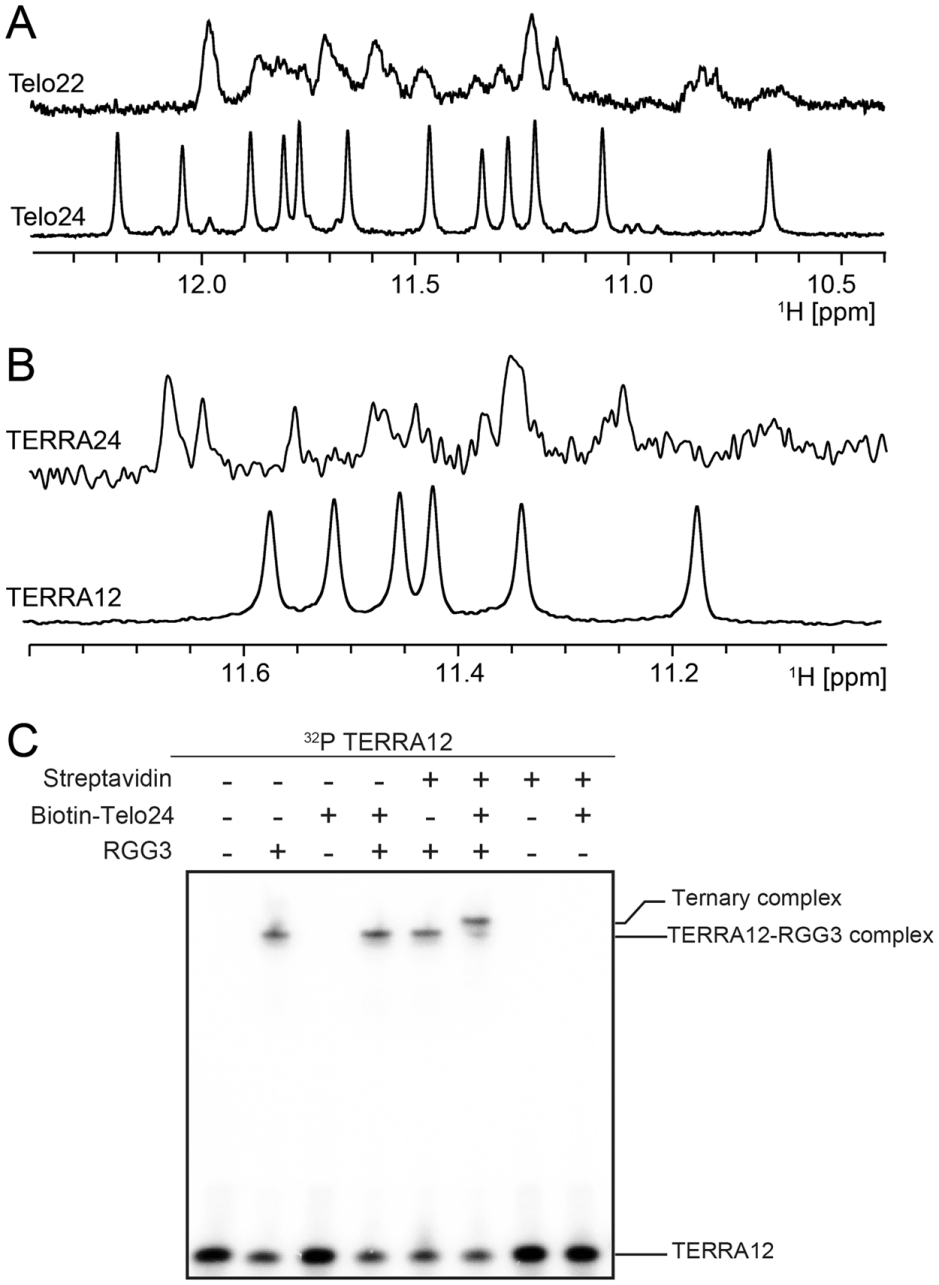
(**A**) The imino proton regions of 1D ^1^H-NMR spectra of Telo22 (top) and Telo24 (bottom). (**B**) The imino proton regions of 1D ^1^H-NMR spectra of TERRA24 (top) and TERRA12 (bottom). (**C**) EMSA of ^32^P-labeled TERRA12 with RGG3, biotinylated Telo24, and streptavidin.

**Figure 3:**
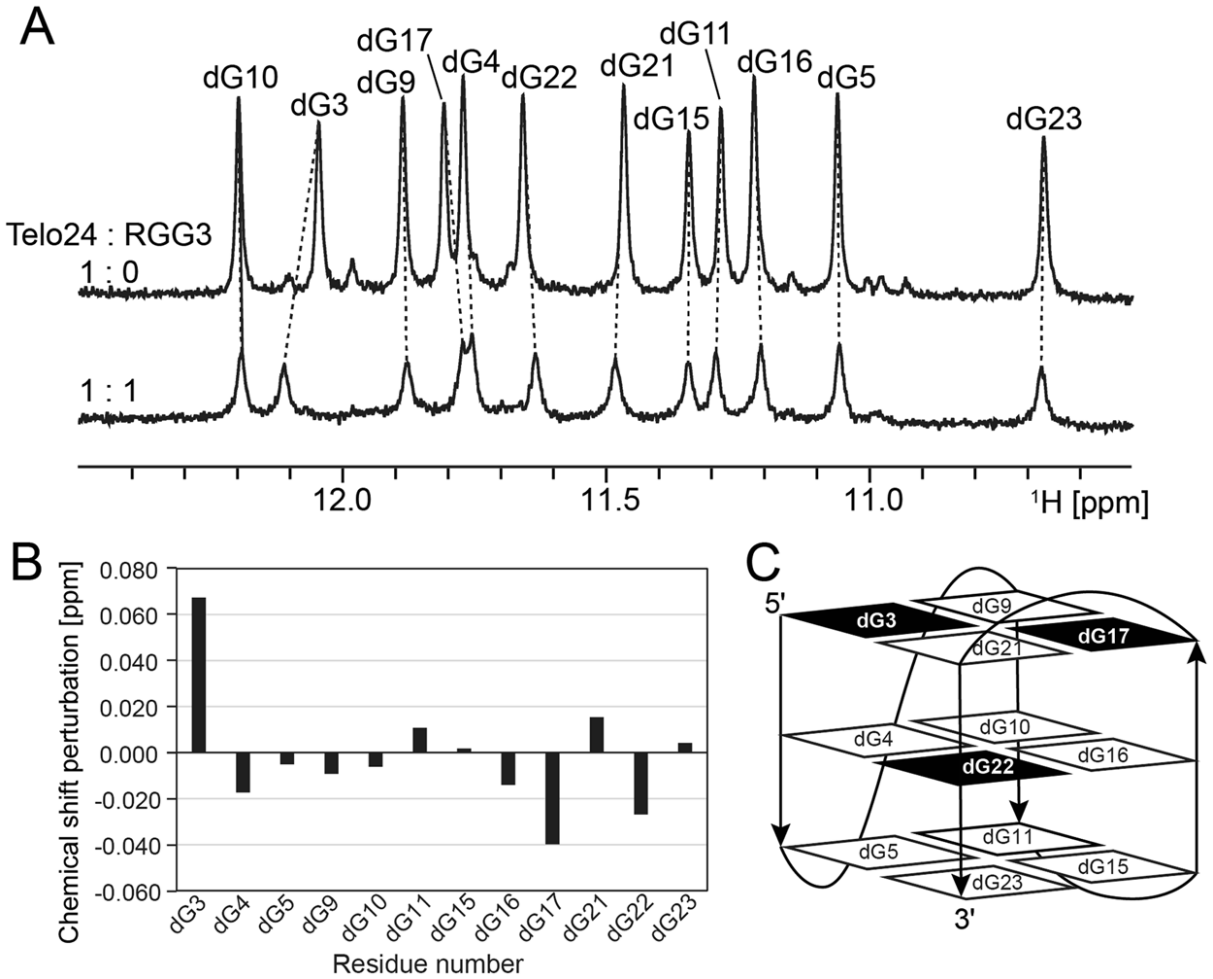
Chemical shift perturbation analysis of Telo24 with RGG3. (**A**) The imino proton regions of 1D ^1^H-NMR spectra of Telo24 either alone (top) or with RGG3 in the ration of 1:1 (bottom). Imino proton resonances were assigned according to the previous report^19^, and are indicated by residue numbers. (**B)** Chemical shift perturbation of each imino proton resonance of Telo24 upon addition of an equivalent amount of RGG3. (**C**) Schematics illustration of the Telo24 G-quadruplex^19^. The guanine residues that exhibited large chemical shift perturbation are highlighted in black.

**Figure 4:**
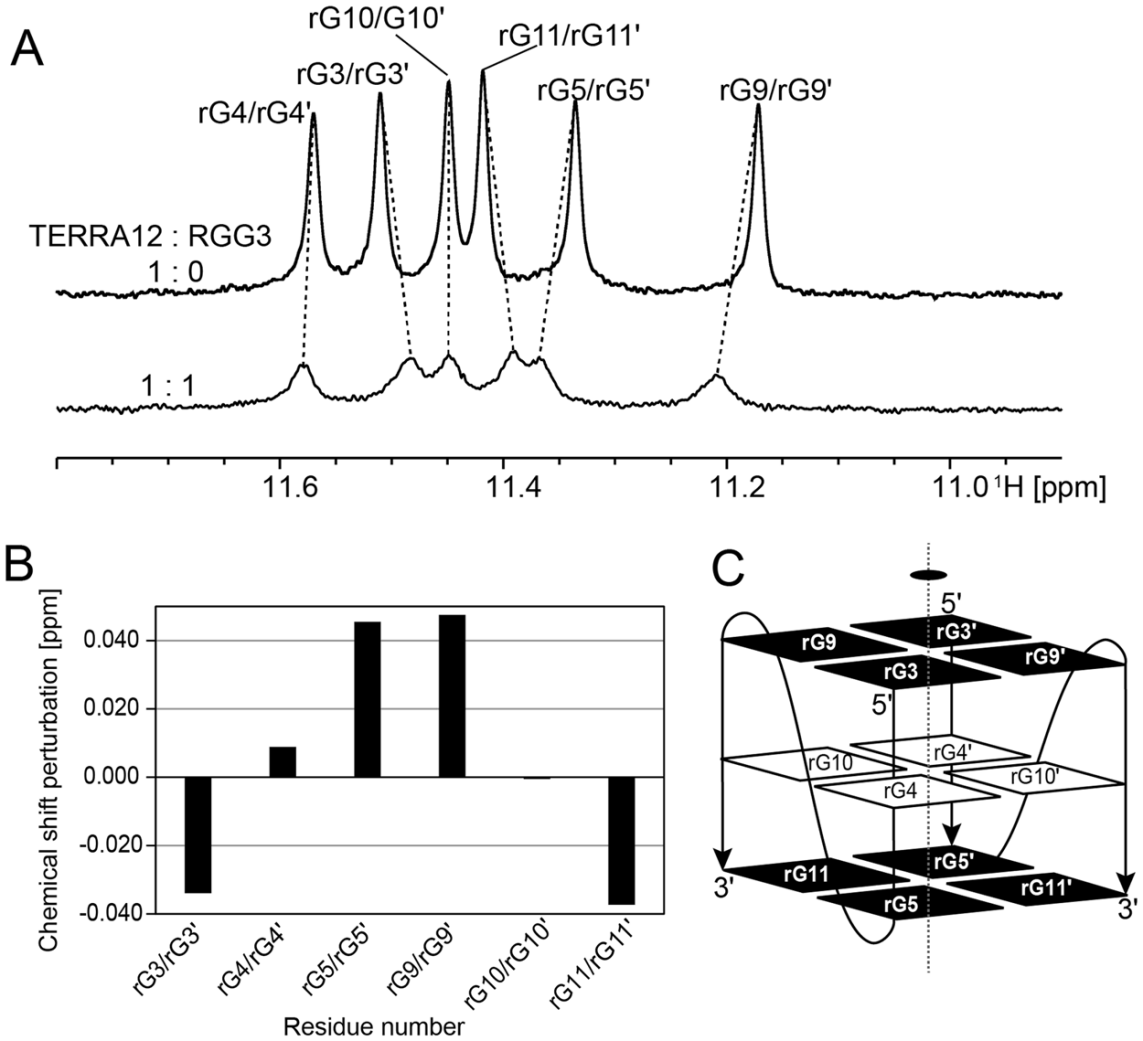
Chemical shift perturbation analysis of TERRA12 with RGG3. (**A**) The imino proton regions of TERRA12 either alone (top) or with RGG3 in the ration of 1:1 (lower). Imino proton resonances were assigned according to the previous report^4^, and are indicated by residue numbers. (**B**) Chemical shift perturbation of each imino proton resonance of TERRA12 upon addition of an equivalent amount of RGG3. (**C**) Schematics illustration of TERRA12 G-quadruplex^4^. The G-quadruplex comprises two monomers. The guanine residues of the second monomer are numbered by prime numbers. The guanine residues that exhibited large chemical shift perturbation are highlighted in black.

G-quadruplex forming Telo22 and TERRA24, which were used in the previous biochemical studies on the interaction with RGG3^8,17,18^, were subjected to NMR analysis. Although many imino proton signals for guanine residues of Telo22 and TERRA24 were observed in 10–12 ppm of ^1^H NMR spectra, which are indicative of G-quadruplex structures, the total number of signals exceeded the number expected for a single G-quadruplex structure, suggesting structural heterogeneity (Fig. 2A,B). Additionally, the signals showed intensity variations, which also supported this suggestion. Such structural heterogeneity of Telo22 and TERRA24 was thought to make further analysis difficult. Therefore, we tested alternative sequences for telomeric DNA and TERRA, Telo24 and TERRA12, respectively. Telo24 is a modified telomeric sequence, which reportedly forms a homogeneous (3 + 1) G-quadruplex structure in the presence of K^+^^19^, as schematically drawn in Fig. 3C. We confirmed the presence of a single G-quadruplex structure on the basis of the observation of twelve imino proton signals (Fig. 2A, bottom), as were observed in the previous report^19^. TERRA12 reportedly forms a homodimer, which takes on a propeller-type parallel-stranded G-quadruplex structure in the presence of K^+^^4^, as schematically drawn in Fig. 4C. The bottom of Fig. 2B shows the imino proton spectrum of TERRA12; the chemical shifts of the imino protons were confirmed to be identical to those in the previous report^4^. Although there are twelve imino protons in the homodimeric TERRA12, only six imino proton signals were observed, because of its dyad symmetry (Fig. 2B).

To investigate the RGG3-binding ability of Telo24 and TERRA12, a fluorescence anisotropy experiment was performed. The addition of aliquots of RGG3 to Telo24-FITC resulted in an increase in the fluorescence anisotropy value of Telo24-FITC, reflecting the formation of the RGG3:Telo24-FITC complex (Fig. S1A). The dissociation constant (*K*_d_) for the RGG3:Telo24-FITC complex was estimated by curve-fitting analysis of the saturation curve to be 46 nM. Likewise, interaction of TERRA12 and RGG3 was also demonstrated by a fluorescence anisotropy experiment involving FITC-TERRA12, the dissociation constant of the RGG3:FITC-TERRA12 complex being estimated to be 31 nM (Fig. S1B).

We next investigated the formation of a ternary complex of Telo24 and TERRA12 with RGG3 by EMSA (Fig. 2C). A shift of the ^32^P-labeled TERRA12 band was observed when RGG3 was added, indicating the formation of a RGG3:TERRA12 binary complex. The band of ^32^P-labeled TERRA12 did not shift on the addition of biotinylated Telo24 if RGG3 was not present, ruling out direct interaction between TERRA12 and Telo24. Biotinylated Telo24 was then added to the RGG3:TERRA12 binary complex, but no further shift was observed. This lack of a change in the mobility may be due to the fact that the molecular weight difference between the binary and ternary complexes, ca. 7.6 k, was not large enough to be detected on EMSA. The same phenomenon was previously observed in EMSA where Telo22 and TERRA24 were used^8^. To evaluate this notion, we subsequently added streptavidin (molecular weight of ca. 70 k). As expected, we observed a super-shift of the band, which indicates that indeed a biotinylated Telo24:RGG3:TERRA12 complex had been formed. We also confirmed that streptavidin itself does not cause a super-shift of the band of the RGG3:TERRA12 binary complex in the absence of biotinylated Telo24. These results demonstrated that Telo24 and TERRA12 have the ability to form a ternary complex with RGG3, like Telo22 and TERRA24 did.

As described above, the clear appearance of imino proton signals of ^1^H NMR spectra for both Telo24 and TERRA12 indicated a formation of homogeneous G-quadruplex structure, respectively. Thus, Telo24 and TERRA12 not only retain RGG3-binding activity equivalent to that of Telo22 and TERRA24, but give ^1^H NMR spectra of higher quality. Therefore, we decided to use Telo24 and TERRA12 for further NMR analysis.

### The nucleic acid residues involved in the formation of the RGG3:telomeric DNA and RGG3:TERRA binary complexes

We performed chemical shift perturbation (CSP) analysis probing imino proton signals to investigate the interaction between RGG3 and either Telo24 or TERRA12, and thereby to find any differences in the manner of interaction. Firstly, RGG3 was added to Telo24 in the molar ratio of 1:1, by which the imino proton signals of guanine residues were perturbed (Fig. 3A). As the imino proton signals of Telo24 were still observed after the addition of RGG3, the interaction of RGG3 with Telo24 did likely not massively disrupt the G-quadruplex structure of Telo24. Significant CSP was observed for dG3 and dG17, both of which constitute the top G-tetrad plane (Fig. 3B,C). The imino proton signal of dG22 in the middle plane exhibited moderate perturbation (Fig. 3B,C). These guanine residues are likely located at the interface with RGG3, although there is the possibility that the CSP of these guanine residues partially reflects a slight rearrangement of the G-quadruplex structure.

Next, CSP analysis of TERRA12 in free and RGG3 bound states was performed. As described previously, the imino proton signals of the same residues in two protomers of the TERRA12 homodimer in the free-state were identical, namely the signals of the corresponding residues were degenerate (e.g., a signal labeled rG3/rG3′ in Fig. 4A). Every signal was still degenerate after addition of RGG3, since the number of signals did not increase. This may be due to averaging of the signals of free and bound states on the NMR chemical shift timescale. The imino proton signals of rG3/rG3′, rG5/rG5′, rG9/rG9′, and rG11/rG11′ of TERRA12 (nucleic acid residues of the second molecule in the homodimer are indicated by prime numbers), which are located in the top or bottom plane of the G-quadruplex structure, were significantly perturbed upon addition of RGG3 to TERRA12 in the molar ratio of 1:1 (Fig. 4A), whereas only small perturbations were observed for the residues in the middle layer. All the known G-quadruplex structures of TERRA are parallel-stranded^4,20,21^. Guanine residues in the top and bottom planes of the parallel-stranded structures are not covered with loops and thus open to the solvent. Therefore, it is reasonable that guanine residues in the top or bottom plane are involved in the interaction with RGG3.

### The amino acid residues of RGG3 involved in the formation of the binary and ternary complexes: the same binding-sites on RGG3 are shared by the binary and ternary complexes

**Figure 5:**
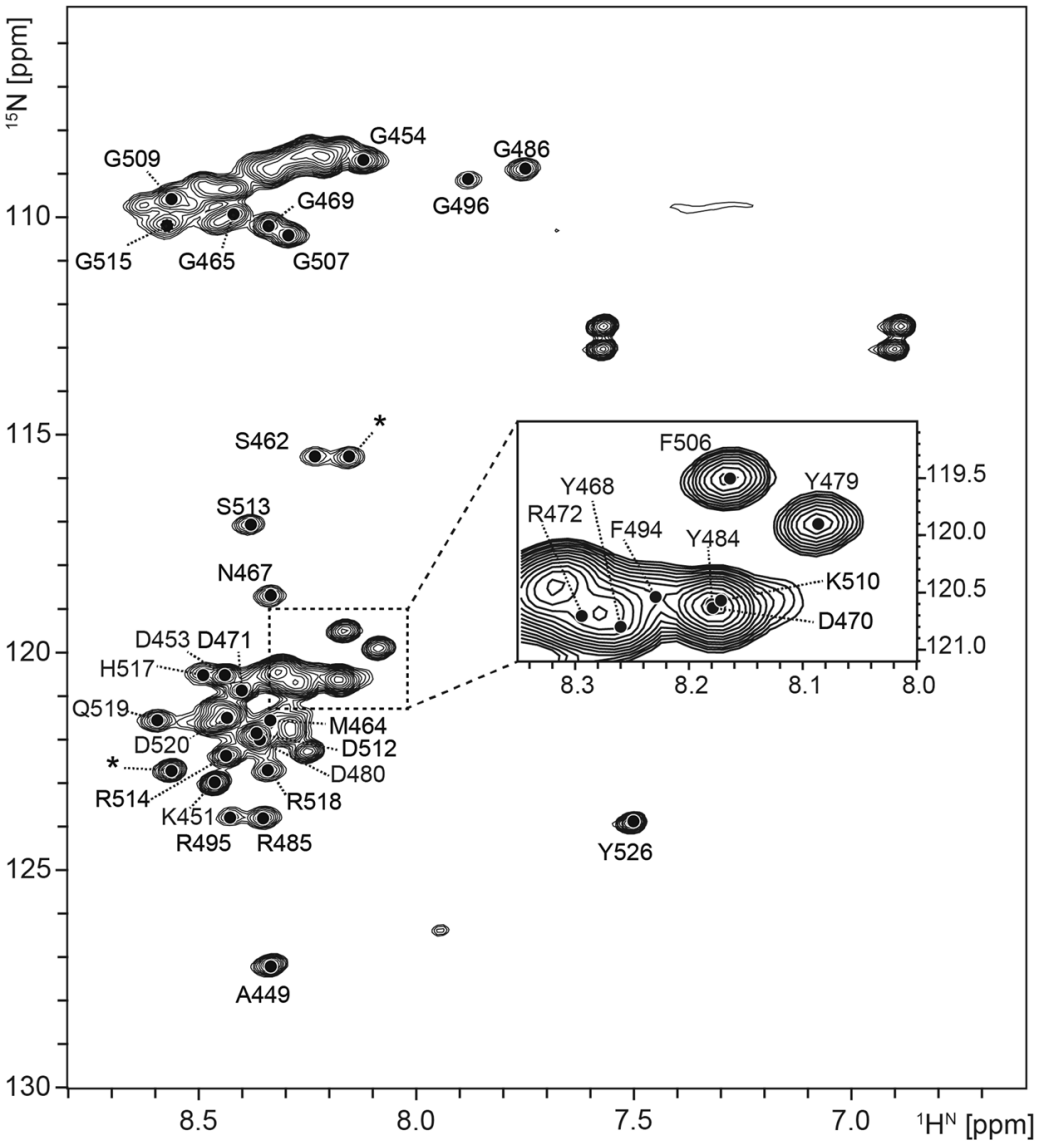
The ^1^H-^15^N HSQC spectrum of RGG3. The assignments of the amide resonances were accomplished on the basis of triple-resonance NMR experiments, and are indicated by residue numbers. The signals indicated by asterisks correspond to the amino acid residues of the GST fragment remaining at the N-terminal region of RGG3.

To identify the amino acid residues of RGG3 at the interaction interface, we firstly assigned the backbone ^1^H,^13^C, and ^15^N signals of ^13^C- and ^15^N-doubly labeled RGG3 using a series of triple-resonance NMR spectra. RGG3, which contains a number of Arg and Gly residues, is known to be intrinsically disordered, having no distinct secondary structure. Generally, the NMR signals of a disordered region are severely overlapped and thus their assignment is difficult. The poor dispersion of the NMR signals of RGG3 indicates that RGG3 is actually disordered. Because of the presence of many Arg and Gly residues, the assignment of Arg and Gly was impossible, however, the residues other than Arg and Gly could be successfully assigned (Fig. 5). An example of the assignments is shown in Fig. S2.

Subsequently, CSP analysis of ^13^C,^15^N-doubly labeled RGG3 was performed by addition of either Telo24, TERRA12, or both. We examined the CSP of the amide signals through ^1^H-^15^N HSQC spectra. The CSP analysis of the amide signals, together with that of imino proton signals in the previous section, confirmed the 1:1 stoichiometries for both the RGG3:telomeric DNA and RGG3:TERRA binary complexes and the 1:1:1 stoichiometry for the ternary complex (data not shown), which are consistent with the previous results obtained by ITC^17^. The amide signals of RGG3 were still overlapped even after the addition of Telo24 and TERRA12 in any combination, suggesting no structure formation. Figures 3 and 4 clearly indicate that RGG3 interacts with both Telo24 and TERRA12. Therefore, we propose that RGG3 does not require an evident structure to recognize the G-quadruplex structures of Telo24 and TERRA12.

**Figure 6:**
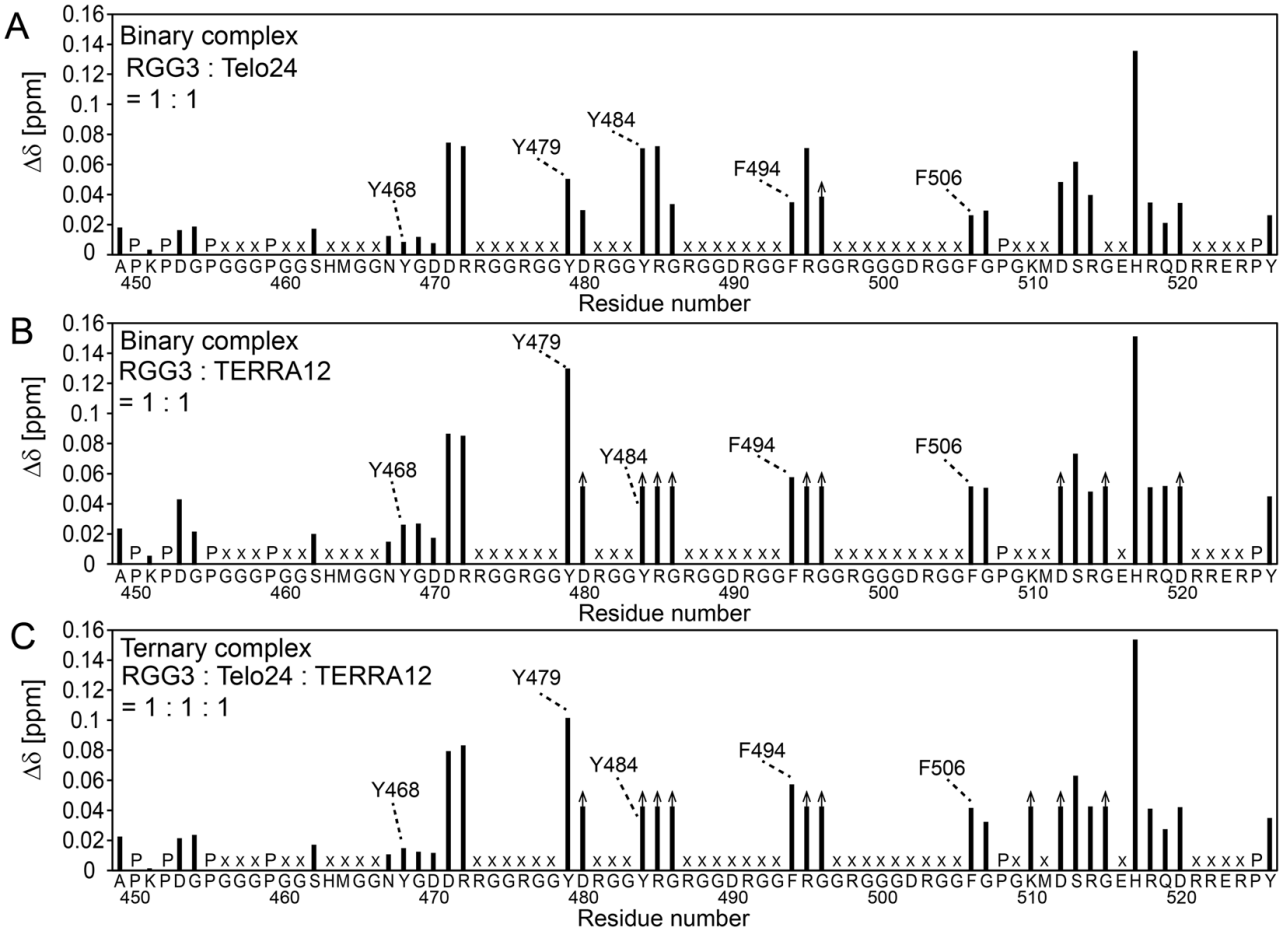
Chemical shift perturbation of the combined amide ^1^H and ^15^N resonances of RGG3 upon addition of an equivalent amount of either Telo24 (**A**), TERRA12 (**B**), or both (**C**). Prolines and unassigned residues are indicated by “P” and “x”, respectively. The residues whose signals disappeared on complex formation due to signal broadening are indicated by arrows.

Large CSP was observed for the assigned residues in the D471-G507 region and those in the C-terminal region of RGG3 (Fig. 6). Surprisingly, the amide signals of Tyr and Phe residues, Y479, Y484, F494, and F506, were commonly perturbed upon addition of either Telo24 or TERRA12 (Fig. 6A,B). It was also noted that a similar CSP profile was observed on the formation of the RGG3:Telo24:TERRA12 ternary complex (Fig. 6C). Thus, Phe and Tyr residues were always involved in the interaction regardless of the kind of complex, which is either the RGG3:Telo24, RGG3:TERRA24, or RGG3:Telo24:TERRA24 complex. Besides, the sequence spanning around residues 510–526 in the C-terminal region of RGG3 is defined as a nuclear localization signal (NLS)^22^. Therefore, Fig. 6 interestingly showed that NLS is also involved in binding with both Telo24 and TERRA24.

### Plastic roles of the Tyr and Phe residues of RGG3 in formation of the binary and ternary complexes

To determine the differences in the mode of interaction between RGG3 with telomeric DNA and that with TERRA in the context of the binary and ternary complexes, the effects of the interaction on the CSP of RGG3 amide signals were analyzed, in particular for Phe and Tyr residues. The assignments of the amide resonances of RGG3 in the binary and ternary complexes were accomplished by means of triple-resonance NMR experiments.

**Figure 7:**
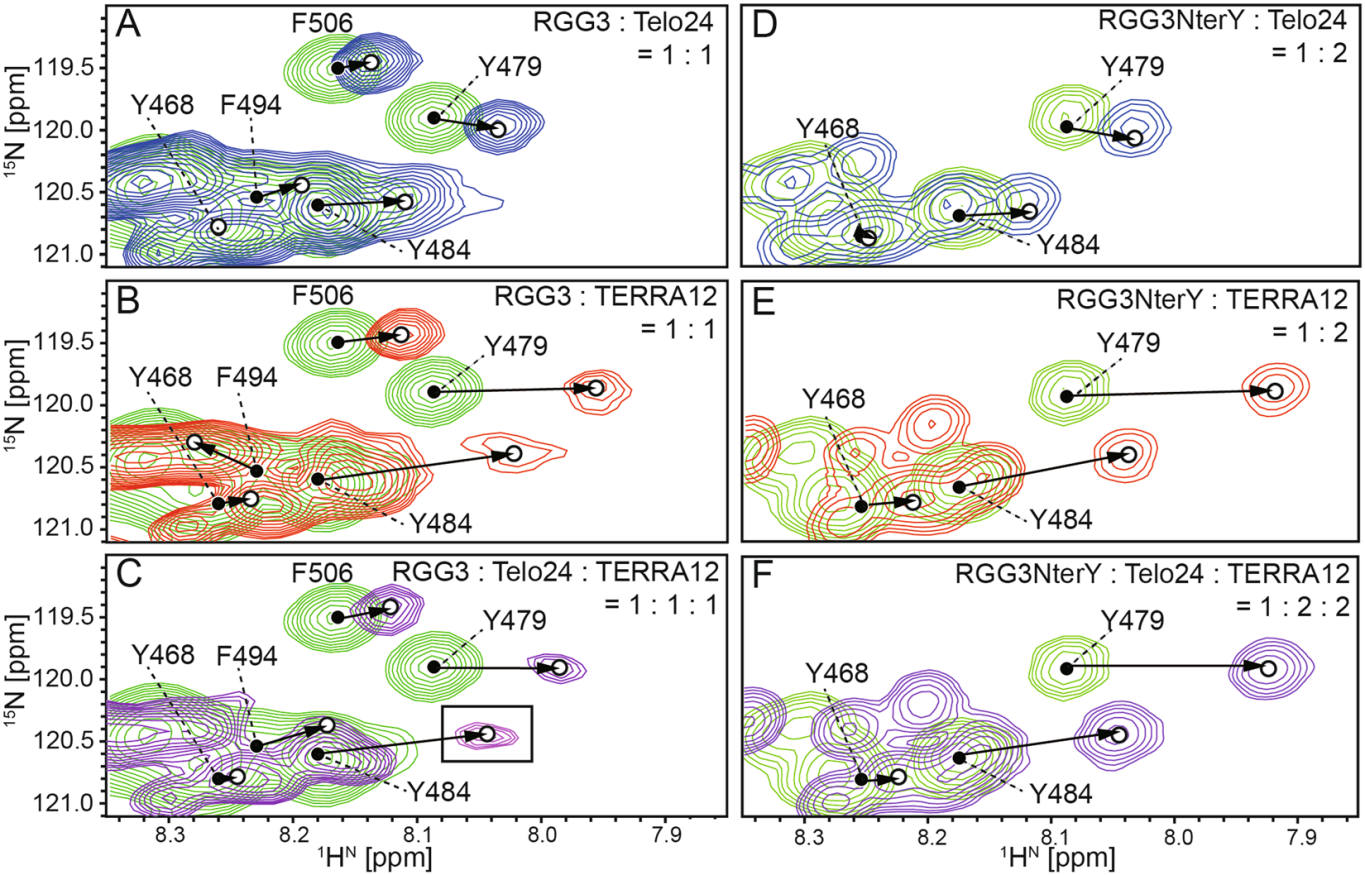
Chemical shift perturbation patterns for the amide ^1^H and ^15^N resonances of tyrosine and phenylalanine residues of either RGG3 or RGG3NterY. An equivalent amount of either Telo24 (**A**), TERRA12 (**B**), or both (**C**) was added to [^13^C, ^15^N]-labeled RGG3. Two equivalent amounts of either Telo24 (**D**), TERRA12 (**E**), or both (**F**) were added to [^13^C, ^15^N]-labeled RGG3NterY. The spectra of free (green) and complex (either blue, red, or purple) RGG3 are overlaid. The amide resonances of tyrosine and phenylalanine residues of RGG3 in its free form (closed circles), and in either the RGG3:quadruplex binary or ternary complex (open circles), which were both assigned by the triple-resonance NMR method, are connected by arrows. An inset in C is shown with 2-fold lower contour level.

Firstly, the positions of the amide signals of F506 in the HSQC spectra for the two binary complexes were compared, and turned out to be almost identical (Fig. 7A,B). On the other hand, the chemical shift of the amide proton of F494 was up-field shifted upon RGG3:Telo24 binary complex formation, whereas it was down-field shifted upon RGG3:TERRA12 binary complex formation (Fig. 7A,B). Since the perturbation was opposite in direction, the effects of the Telo24- and TERRA12-interactions on CSP are easily distinguishable. This difference in CSP should originate from the difference in the interaction modes between RGG3 with the two different G-quadruplex structures of telomeric DNA and TERRA, respectively. Upon formation of the RGG3:Telo24:TERRA12 complex, namely in the presence of both Telo24 and TERRA12, the chemical shift of the amide proton of F494 exhibited an up-field shift as in the case of the RGG3:Telo24 binary complex (Fig. 7C). Therefore, F494 is supposed to interact with Telo24 in the ternary complex.

Next the CSP of Tyr residues in each complex were compared. The chemical shift of the amide proton of Y484 in the RGG3:Telo24 binary complex showed an up-field shift (Fig. 7A). We could not assign the signals of Y484 in either the RGG3:TERRA12 binary complex or ternary complex due to severe broadening (Fig. 7B,C). To overcome this difficulty, we constructed an N-terminal fragment protein of RGG3 (RGG3NterY), which contains only the N-terminal and Tyr-rich regions of RGG3 (Fig. 1B). Since RGG3NterY interacts more weakly with Telo24 and TERRA than RGG3, two equivalent amounts of either Telo24 or TERRA12 were added to RGG3NterY for spectrum comparison. In either case, the Y484 signal of RGG3NterY was trackable and successfully assigned. The chemical shift of the amide proton of Y484 in RGG3NterY exhibited small perturbation upon addition of Telo24 (Fig. 7D), whereas addition of TERRA12 resulted in large chemical shift perturbation (Fig. 7E). These CSP clearly showed that RGG3NterY retained the ability to bind to Telo24 and TERRA12, individually, and that the interaction modes of Y484 with Telo24 and TERRA12 are different. However, when both Telo24 and TERRA12 were added to RGG3NterY, the resultant HSQC spectrum of RGG3NterY was almost the same as that with only TERRA12 added (Fig. 7E,F). This indicates that in the presence of both Telo24 and TERRA12, RGG3NterY does not form the ternary complex, but preferentially forms the binary complex with TERRA12. Then, it was noticed that the chemical shifts of the assigned amide signals of the free RGG3NterY and the RGG3NterY:TERRA12 complex forms were similar to those of the corresponding amide signals of the free RGG3 and the RGG3:TERRA12 complex forms, respectively. Therefore, the interaction mode for the N-terminal half of RGG3 in the RGG3: TERRA12 complex should have been preserved in the RGG3NterY:TERRA12 complex. Based on the assignment of RGG3NterY in the RGG3NterY:TERRA12 complex, we were able to assign the amide signal of Y484 in the RGG3:TERRA12 binary complex, whose signal could not be assigned otherwise due to severe signal-broadening of the triple-resonance spectra (Fig. 7B). Furthermore, a weak signal was also found in the spectrum of the RGG3:Telo24:TERRA12 ternary complex (Fig. 7C). This signal is most likely the amide signal of Y484 in the ternary complex. Note that, the signal found for Y484 in the RGG3:Telo24 binary complex was not observed in the ternary complex. These results suggest that Y484 is involved in the interaction with TERRA12 in the ternary complex.

Additionally, in the case of RGG3, the amide signals of R485 and G486, which are neighboring residues of Y484, disappeared through signal-broadening upon addition of TERRA12, either alone or with Telo24 (data not shown). On the other hand, the amide signals of R485 and G486 in RGG3 did not disappear upon addition of Telo24 alone. Thus, it is suggested that not only Y484 but also its neighboring residues are involved in the interaction with TERRA12 in the ternary complex.

In the case of Y479 in RGG3, the chemical shift of the amide proton was slightly perturbed toward up-field upon addition of Telo24 (Fig. 7A), while the addition of TERRA12, either alone or together with Telo24, resulted in large perturbation toward up-field (Fig. 7B,C). These results indicate that Y479 is involved in the interaction with TERRA12 in the ternary complex.

Finally, the chemical shift of the amide proton of Y468 in RGG3 exhibited small perturbation upon addition of either Telo24 or TERRA12 (Fig. 7A,B). Similarly, the CSP for the Y468 signal upon the formation of the RGG3:Telo24:TERRA12 ternary complex was small (Fig. 7C), therefore, Y468 seems not to be involved in the interaction with Telo24 and TERRA12.

Additionally, CSP analysis of RGG3 was also performed with Telo22 and TERRA24. The CSP patterns for Phe and Tyr residues with Telo22 and TERRA24 were very similar to those in the experiments with Telo24 and TERRA12 (Fig. S3). In brief, for a Phe residue, F494, the CSP pattern for the RGG3:Telo22:TERRA24 ternary complex was similar to that for the RGG3:Telo22 binary complex. On the contrary, for two Tyr residues, Y479 and Y484, The CSP patterns for the ternary complex were similar to those for the RGG3:TERRA24 binary complex. Therefore, these Phe and Tyr residues are supposed to interact with Telo22 and TERRA24, respectively, in the ternary complex. These results suggest that the modes of the interactions for the RGG3:Telo24:TERRA12 ternary complex are basically the same for the RGG3:Telo22:TERRA24 complex on which biochemical studies were carried out previously^8,17,18^.

Overall, F494, Y479, and Y484 can play plastic roles in the recognition depending on their binding partner. F494 preferentially interacts with telomeric DNA, whereas Y479 and Y484 preferentially interact with TERRA in the ternary complex, although these residues interact with either telomeric DNA or TERRA in each binary complex. As the guanine bases, which form G-tetrads, are located in the core of the G-quadruplex structure, while the sugar-phosphate backbone moieties are mainly located outside, we assume the possible binding mechanisms as follows: the non-polar phenylalanine side chain of RGG3 may preferably interact with the non-polar 2′-methylene group of the deoxyribose of telomeric DNA through hydrophobic interaction in the ternary complex. On the other hand, the hydroxyl group of the tyrosine side chain of RGG3 may preferably interact with the 2′-hydroxy group of the ribose of TERRA through hydrogen bonding in the ternary complex.

## Conclusion

In the current study, we investigated the interaction between TLS RGG3 and either one or both of telomeric DNA and TERRA. We identified the amino acid residues of TLS RGG3, and guanine residues of telomeric DNA and TERRA that are involved in the formation of the complex. We then found that TLS RGG3 interacts mainly with the top plane of the G-tetrad of the G-quadruplex structure formed by telomeric DNA, while in TERRA, the top and bottom G-tetrad planes are the binding sites for TLS RGG3. It was also evident that the binding of telomeric DNA and TERRA does not induce the formation of secondary structures of TLS RGG3.

Based on our previous functional mutation analysis, the Phe and Tyr residues of TLS RGG3 were expected to individually interact with telomeric DNA and TERRA^18^. To validate this, we firstly performed CSP analysis, where either telomeric DNA or TERRA was titrated against TLS RGG3. The results were rather surprising. It was revealed that not only the Phe residues but also the Tyr residues are involved in the interaction with telomeric DNA in the RGG3:telomeric DNA binary complex. Likewise, not only the Tyr residues but also the Phe residues were shown to be involved in the interaction with TERRA in the RGG3:TERRA binary complex. Thus, both Phe and Tyr residues were capable of binding to both telomeric DNA and TERRA. We then noticed that each of Y479, Y484, and F494, and their neighboring residues showed different CSP patterns depending on which binary complex RGG3 was in, indicating that these residues exhibit different binding-modes upon binding with telomeric DNA and TERRA.

Subsequently, we performed CSP analysis of the formation of the RGG3:telomeric DNA:TERRA ternary complex. It was shown for F494 that the CSP pattern for the formation of the ternary complex is the same as that for the RGG3:telomeric DNA binary complex, suggesting that the interaction between F494 and telomeric DNA in the binary complex is retained in the ternary complex. This suggests that F494 can play plastic roles: F494 recognizes telomeric DNA in both the RGG3:telomeric DNA binary and ternary complexes, while in the RGG3:TERRA binary complex, F494 just assists the overall binding.

For Y479 and Y484 on the other hand, the CSP patterns for the formation of the RGG3:telomeric DNA:TERRA ternary complex are the same as that for the RGG3:TERRA binary complex. This indicates that Y479 and Y484 also can play plastic roles: they recognize TERRA in both the RGG3:TERRA binary and ternary complexes, while in the RGG3:telomeric DNA binary complex, they play an assisting role in the overall binding.

**Figure 8:**
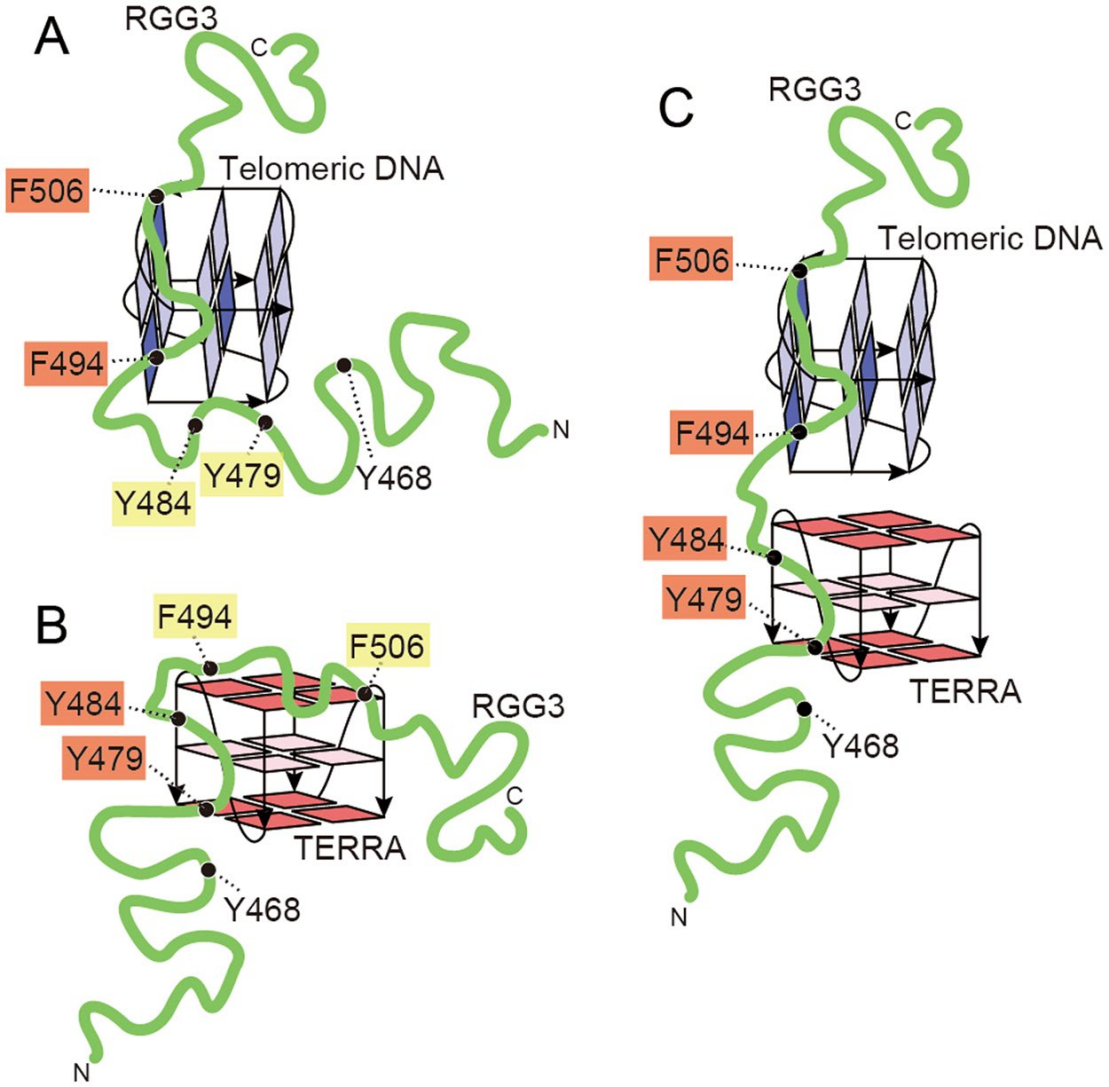
Models of RGG3 in a complex with the G-quadruplex of either telomeric DNA (**A**), TERRA12 (**B**), or both (**C**). The phenylalanine and tyrosine residues indicated by orange shading are recognizing the primary quadruplexes, while the residues indicated by yellow shading are assisting the binding.

By integrating the observations made on the CSP analysis, we propose a model of the binary and ternary complexes formed by TLS RGG3, telomeric DNA, and TERRA (Fig. 8). The disordered RGG3 winds around the binding sites of the G-quadruplexes formed by telomeric DNA and TERRA. TLS RGG3 is suggested to be flexible enough to place residues Y479, Y484, and F494, which can play plastic roles, on the binding sites on the G-quadruplexes of telomere DNA and TERRA depending on which complex needs to be formed. The involvement of Phe, Tyr, and neighboring residues of RGG3 in binding to a quadruplex in each binary complex contributes to increase the affinity, resulting in the efficient formation of each binary complex. Then, this increases the chance of the formation of a ternary complex. When the ternary complex is formed, these residues plastically change their target quadruplex: Phe and neighboring residues bind to the telomeric DNA quadruplex, while Tyr and neighboring residues bind to the TERRA quadruplex. Thus, these residues plastically change their roles for the efficient formation of the ternary complex, which would be biologically relevant. This would be how an intrinsically disordered domain, RGG3 of TLS, can effectively play key roles in ternary complex formation. Eventually, ternary complex formation would lead to recruitment of histone-modifying enzymes to telomere and thus telomere shortening through the inhibition of telomerase-independent telomere-elongation caused by homologous recombination of telomeric DNA.

## Methods

### Preparation of G-quadruplexes

24-nt d[TTGGG(TTAGGG)_3_A] (Telo24) and 22-nt d[AGGG(TTAGGG)_3_] (Telo22) were used as telomeric DNA. 24-nt r(UUAGGG)_4_ (TERRA24) and 12-nt r(UAGGGUUAGGGU) (TERRA12) were used as TERRA (Table 1). All of the above nucleic acids, which were synthesized, purified by high-performance liquid chromatography, and desalted, were purchased from FASMAC (Japan). 1 mM Telo24, Telo22, and TERRA24, and 2 mM TERRA12 were annealed by incubation at 95 °C for 10 min, followed by cooling to 20 °C at a rate of ca. 1 °C/min in 20 mM potassium phosphate buffer (pH 6.2) containing 50 mM KCl. As the G-quadruplex structure of TERRA12 comprises a homodimer, the annealing of 2 mM TERRA12 yields 1 mM G-quadruplex.

**Table 1:** Sequences of telomeric DNA and TERRA used in this study.

Name	Sequence
Telo22	d[AGGG(TTAGGG)_3_]
Telo24	d[TTGGG(TTAGGG)_3_A]
TERRA24	r(UUAGGG)_4_
TERRA12	r(UAGGGUUAGGGU)
Telo24-FITC	d[TTGGG(TTAGGG)_3_A]-FITC
FITC-TERRA12	FITC-r(UAGGGUUAGGGU)

### Protein preparation

The gene encoding RGG3 of TLS was subcloned into the pGEX6P-1 vector by the method previously described (pGEX-RGG3)^8^. The plasmid for expressing the N-terminal and Tyr-rich regions of RGG3 (RGG3NterY, Fig. 1A) was prepared by polymerase chain reaction (PCR) using the pGEX-RGG3 as a template. d(GGACTAATAGCGTGGAGGCTTCCGA) and d(CCACGCTATTAGTCCCCGCCGCGGC) were used as forward and reverse primers, respectively. The primers were designed to insert a stop codon between the codons corresponding to D490 and R491. RGG3 was expressed and purified by the following procedure. *Escherichia coli* BL21 cells were transformed with pGEX-RGG3. Expression of non-labeled and isotope-labeled RGG3 was performed in LB medium and minimal (M9) medium, respectively. For ^13^C- and ^15^N-labeling, 2 g/L ^13^C-glucose and 1 g/L ^15^N-ammonium chloride were used as carbon and nitrogen sources, respectively. The culture was incubated at 37 °C, protein expression was induced with 0.2 mM β-D-1-thiogalactopyranoside (IPTG) when OD_600_ reached 0.7, and then the culture was further incubated at 25 °C for 16 h. The cells from 1 L culture were harvested by centrifugation and resuspended in lysis buffer, which was 100 mM Tris-HCl buffer (pH 7.5) containing 500 mM NaCl and 1 mM dithiothreitol (DTT). After addition of 10 mM benzamidine hydrochloride, 5 U/mL DNase I (Invitrogen, US), 1 mg/L RNase (Nippon Gene, Japan), and 0.2 g/L lysozyme, the cells were lysed by sonication at 4 °C. The lysate was supplemented with 1 mM EDTA and 0.1% Triton X-100, and then centrifuged to obtain a supernatant. Subsequently, GST-fused RGG3 in the supernatant was trapped on a 2 mL bed volume of glutathione-sepharose 4B resin (GE Healthcare, US). The protein bound resin was washed thoroughly with lysis buffer. GST was then cleaved on-column by addition of 80 U Prescision protease (GE Healthcare, US) and further incubation at 4 °C for 16 h. The detached RGG3 was eluted and collected from the resin. The collected RGG3 was further purified by cation exchange chromatography under the following conditions: column, HiTrap SP HP (1 mL, GE Healthcare, US); temperature, 4 °C; mobile phase to collect flow through, 100 mM Tris-HCl (pH 7.8); mobile phase for elution, NaCl linear gradient from 0 to 1 M in 100 mM Tris-HCl (pH 7.8) taking 100 min; flow rate, 0.5 mL/min; and detection, A_280_. The purified protein, which was collected as the major fraction, was dialyzed against 20 mM potassium phosphate buffer (pH 6.2) containing 50 mM KCl and 1 mM DTT. Four mutants of RGG3 were also expressed and purified in the same way. Similarly, non-labeled and isotope-labeled RGG3NterY were expressed and purified by this procedure, except that the cation exchange chromatography was performed at pH 7.2.

### NMR spectroscopy

All NMR samples were prepared in 20 mM potassium phosphate buffer (pH 6.2) containing 50 mM KCl, 1 mM DTT, 0.1 mM 2,2-dimethyl-2-silapentane-5-sulfonate (DSS), and 5% D_2_O. ^1^H spectra of Telo24, Telo22, TERRA12, and TERRA24 were recorded at the concentration of 75 μM. ^1^H spectra after the addition of non-labeled RGG3 to either Telo24 or TERRA12 in the molar ratio of 1:1 were also recorded. ^1^H-^15^N HSQC, ^1^H-^13^C HSQC, HNCO, HN(CA)CO, HNCACB, and CACB(CO)NH spectra of ^13^C- and ^15^N-doubly labeled RGG3 or RGG3NterY were recorded at the concentration of 300 μM, and used for the assignment of backbone ^1^H, ^15^N, and ^13^C resonances. For the chemical shift perturbation (CSP) experiment, either Telo24, TERRA12, or both were added to RGG3 or RGG3NterY in the molar ratio of 1:1 or 1:1:1. The backbone ^1^H, ^13^C, and ^15^N resonances of RGG3 and RGG3NterY were then assigned by the same method as that described above. All the above NMR data were acquired at 298 K with Bruker DRX 600 and AVANCE III HD 600 MHz NMR spectrometers equipped with a cryoprobe. DSS was used as the ^1^H chemical shift reference, and the ^13^C and ^15^N chemical shifts were calibrated indirectly using DSS via their gyromagnetic ratios. NMR data were processed with NMRPipe/NMRDraw^23^ or TopSpin/XWIN-NMR (Bruker Biospin, Japan). Spectral analysis was performed with MagRO-NMRView^24,25^ and Sparky (http://www.cgl.ucsf.edu/home/sparky/). Chemical shift perturbations, Δδ, for amide resonances were calculated using the following equation; Δδ = [(Δδ_H_)^2^ + (Δδ_N_/6.5)^2^]^1/2 ^^26^, where Δδ_H_ and Δδ_N_ are the chemical shift differences for ^1^H^N^ and ^15^N resonances between before and after the addition of either Telo24, TERRA12, or both. The value 6.5 is a chemical shift scaling factor, which is a ratio of the average variances of the amide nitrogen and proton chemical shifts for the 20 amino acids in various proteins.

### Fluorescent anisotropy experiment

Telo24 conjugated with fluorescein isothiocyanate (FITC) at the 3′ end (Telo24-FITC) and TERRA12 conjugated with FITC at the 5′ end (FITC-TERRA12) were purchased from FASMAC (Japan) (Table 1). Telo24-FITC and FITC-TERRA12 were prepared by the same annealing method as that described above. 450 nM Telo24-FITC or FITC-TERRA12 was titrated with non-labeled RGG3 in a molar ratio from 1:0 to 1:3. At each titration step, fluorescent anisotropy was recorded with a JASCO spectra manager FP-8500 at an excitation wavelength of 490 nm and an emission wavelength of 520 nm at 20 °C.

### Electrophoretic mobility shift assaying with streptavidin

1 μM ^32^P-labeled TERRA12 and biotinylated Telo24 were annealed by incubation at 95 °C, followed by cooling to 4 °C at a rate of ca. 2 °C/min in 50 mM Tris-HCl buffer (pH 7.5) containing 100 mM KCl. Binding reactions were performed in a final volume of 20 μL using 20 fmol of the ^32^P-labeled TERRA12 in a binding buffer (50 mM Tris-HCl buffer (pH 7.5), 100 mM KCl, 0.5 mM EDTA, 0.5 mM DTT, 0.2 mg/mL BSA, and 1 μg/mL calf thymus DNA) with or without either 250 nM RGG3, biotinylated-Telo24, or 100 nM streptavidin. Samples were incubated for 1 h at 20 °C, and then loaded onto an 8% polyacrylamide (acrylamide/bisacrylamide, 19:1) nondenaturing gel. The gel and the electrophoresis buffer contained 0.5× TBE buffer (45 mM Tris base, 45 mM boric acid, and 0.5 mM EDTA) including 20 mM KCl. Electrophoresis was performed at 10 V/cm for 2 h at 4 °C. The gel was exposed in a phosphorimager cassette and imaged (Personal Molecular Imager FX, Bio-Rad, Hercules, CA).

## Electronic supplementary material

Supporting information

## References

[1] Moyzis RK (1988). A highly conserved repetitive DNA sequence, (TTAGGG)n, present at the telomeres of human chromosomes. Proc. Natl. Acad. Sci. USA.

[2] Azzalin CM, Reichenbach P, Khoriauli L, Giulotto E, Lingner J (2007). Telomeric repeat containing RNA and RNA surveillance factors at mammalian chromosome ends. Science.

[3] Schoeftner S, Blasco A (2008). Developmentally regulated transcription of mammalian telomeres by DNA-dependent RNA polymerase II. Nature Cell Biol..

[4] Martadinata H, Phan AT (2009). Structure of propeller-type parallel-stranded RNA G-quadruplexes, formed by human telomeric RNA sequences in K^+^ solution. J. Am. Chem. Soc..

[5] Zahler AM, Willliamson JR, Cech TR (1991). Prescott, D. M. Inhibition of telomerase by G-quartet DNA structures. Nature.

[6] Zaug AJ, Podell ER, Cech TR (2005). Human POT1 disrupts telomeric G-quadruplexes allowing telomerase extension *in vitro*. Proc. Natl. Acad. Sci. USA.

[7] Lipps HJ, Rhodes D (2009). G-quadruplex structures: *in vivo* evidence and function. Trends Cell Biol..

[8] Takahama K (2013). Regulation of telomere length by G-quadruplex telomere DNA- and TERRA-binding protein TLS/FUS. Chem. Biol..

[9] Crozat A, Åman P, Mandahl N (1993). Fusion of CHOP to a novel RNA-binding protein in hyman myxoid liposarcoma. Nature.

[10] Kanai Y, Dohmae N, Hirokawa N (2004). Kinesin transports RNA: isolation and characterization of an RNA-transporting granule. Neuron.

[11] Wang X (2008). Induced ncRNAs allosterically modify RNA-binding proteins in cis to inhibit transcription. Nature.

[12] Yang L, Embree LH, Tsai S, Hickstein DD (1998). Oncoprotein TLS interacts with serine-arginine proteins involved in RNA splicing. J. Biol. Chem..

[13] Ratti A, Buratti E (2016). Physiological functions and pathobiology of TDP-43 and FUS/TLS proteins. J. Neurochem..

[14] Deng Z, Norseen J, Wiedmer A, Riethman H, Lieverman PM (2009). TERRA RNA binding to TRF2 facilitates heterochromatin formation and ORC recruitment at telomeres. Mol. Cell..

[15] Benetti R (2007). Suv4-20h deficiency results in telomere elongation and derepression of telomere recombination. J. Cell. Biol..

[16] Arnoult N, Van Beneden A, Decottignies A (2012). Telomere length regulates TERRA levels through increased trimethylation of telomeric H3K9 and HP1α. Nat. Struct. Mol. Biol..

[17] Takahama K, Oyoshi T (2013). Specific binding of modified RGG domain in TLS/FUS to G-quadruplex RNA: tyrosines in RGG domain recognize 2′-OH of the riboses of loops in G-quadruplex. J. Am. Chem. Soc..

[18] Takahama K (2015). G-quadruplex DNA- and RNA-specific-binding proteins engineered from the RGG domain of TLS/FUS. ACS Chem. Biol..

[19] Luu KN, Phan AT, Kuryavyi V, Lacroix L, Patel DJ (2006). Structure of the human telomere in K^+^ solution: an intramolecular (3 + 1) G-quadruplex scaffold. J. Am. Chem. Soc..

[20] Xu Y, Kaminaga K, Komiyama M (2008). G-quadruplex formation by human telomeric repeats-containing RNA in Na^+^ solution. J. Am. Chem. Soc..

[21] Martadinata H, Phan AT (2013). Structure of human telomeric RNA (TERRA): stacking of two G-quadruplex blocks in K^+^ solution. Biochemistry.

[22] Dormann D (2010). ALS-associated fused in sarcoma (FUS) mutations disrupt transportin-mediated nuclear import. EMBO J..

[23] Delaglio F (1995). NMRPipe: a multidimensional spectral processing system based on UNIX pipes. J. Biomol. NMR.

[24] Kobayashi N (2007). KUJIRA, a package of integrated modules for systematic and interactive analysis of NMR data directed to high-throughput NMR structure studies. J. Biomol. NMR.

[25] Kobayashi N (2012). An automated system designed for large scale NMR data deposition and annotation: application to over 600 assigned chemical shift data entries to the BioMagResBank from the Riken Structural Genomics/Proteomics Initiative internal database. J. Biomol. NMR.

[26] Mulder FAA, Shipper D, Bott R, Boelens R (1999). Altered flexibility in the substrate-binding site of related native and engineered high-alkaline bacillus subtilisins. J. Mol. Biol..

