# Supplementary material for: Plastic roles of phenylalanine and tyrosine residues of TLS/FUS in complex formation with the G-quadruplexes of telomeric DNA and TERRA
Source: Sci Rep. 2018 Feb 12;8:2864. doi: 10.1038/s41598-018-21142-1 (PMC5809584; doi:10.1038/s41598-018-21142-1)
Supplement: Supplementary file 1 — Supporting information [file 41598_2018_21142_MOESM1_ESM.pdf]

Plastic roles of phenylalanine and tyrosine residues of TLS/FUS in complex formation with the G-quadruplexes of telomeric DNA and TERRA

Keiko Kondo[1], Tsukasa Mashima[1,2], Takanori Oyoshi[3], Ryota Yagi[3], Riki Kurokawa[4], Naohiro Kobayashi[5], Takashi Nagata[1,2], Masato Katahira[1,2,*]

Supporting information

THE ASSIGNMENTS

A conventional backbone assignment procedure was performed for RGG3 in the free form using HNCO, HN(CA)CO CACB(CO)NH, and HNCACB spectra, by which $^{1}H^{N}$, ^{15}N, $^{13}C\alpha$, $^{13}C\beta$, and ^{13}CO chemical shifts were obtained. Because of the inherent sequence redundancy of the Arg-Gly-rich region, the assignment of the residues within this region is considered to be challenging. However, the residues other than Arg and Gly could be successfully assigned. As an example, the assignments of F494, R495, and G496 are demonstrated in Figure S2.

FIGURE LEGENDS

Figure S1. The evaluation of equilibrium binding of RGG3 to either Telo24-FITC (A) or FITC-TERRA12 (B) by fluorescence anisotropy experiments. The difference in the fluorescence anisotropy value between before and after the addition of RGG3 were calculated at each titration step, and then normalized.

Figure S2. Sequential connectivities of $^{13}C\alpha$ and $^{13}C\beta$ chemical shifts for F494, R495, and G496 of RGG3, which were achieved by backbone assignment procedure using HNCACB and CBCA(CO)NH spectra.

Figure S3. Chemical shift perturbation patterns for the amide ^{1}H and ^{15}N resonances of tyrosine and phenylalanine residues or RGG3 on the addition of an equivalent amount of either Telo22 (A), TERRA24 (B), or both (C). The spectra of free (green) and complex (either blue, red or purple) RGG3 are overlaid. The amide resonances of tyrosine and phenylalanine residues of RGG3 in free form (closed circles), and in either the binary or ternary complex (open circles), which were both assigned based on the assignment made in Figure 7, are connected by arrows. The signals of amino acid residues with asterisks were broadened and disappeared on complex formation.

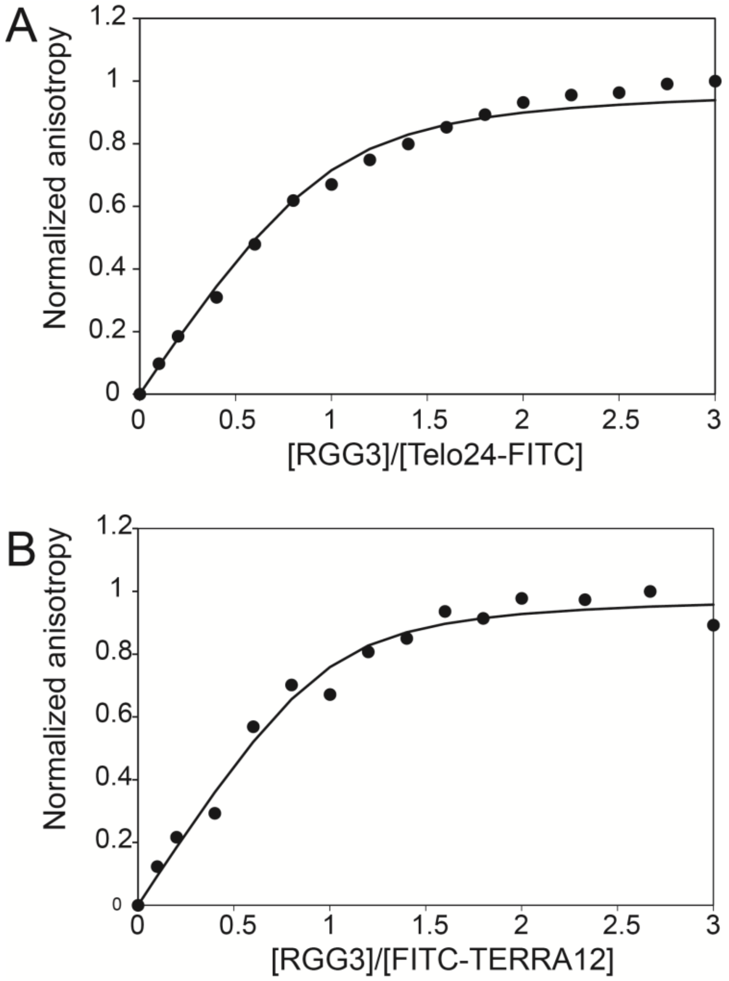
A
Normalized anisotropy
1.2
1
0.8
0.6
0.4
0.2
0
0.5
1.5
2
2.5
3
[RGG3]/[Telo24-FITC]
B
Normalized anisotropy
[RGG3]/[FITC-TERRA12]

Figure S1

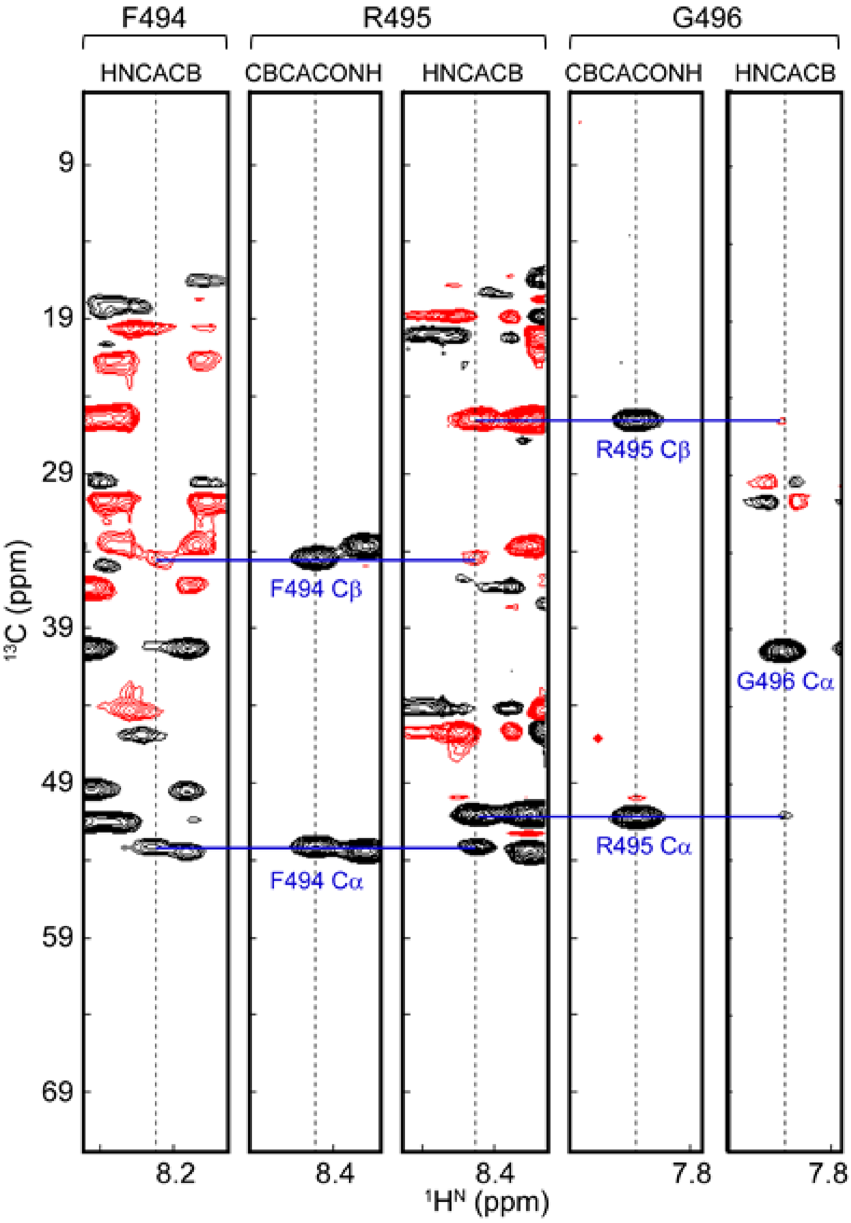
F494
R495
G496
HNCACB
CBCACONH
HNCACB
CBCACONH
HNCACB
9
19
29
39
49
59
69
^{13}C (ppm)
R495 Cβ
F494 Cβ
G496 Cα
R495 Cα
F494 Cα
8.2
8.4
8.4
7.8
7.8
^{1}H^{N} (ppm)

Figure S2

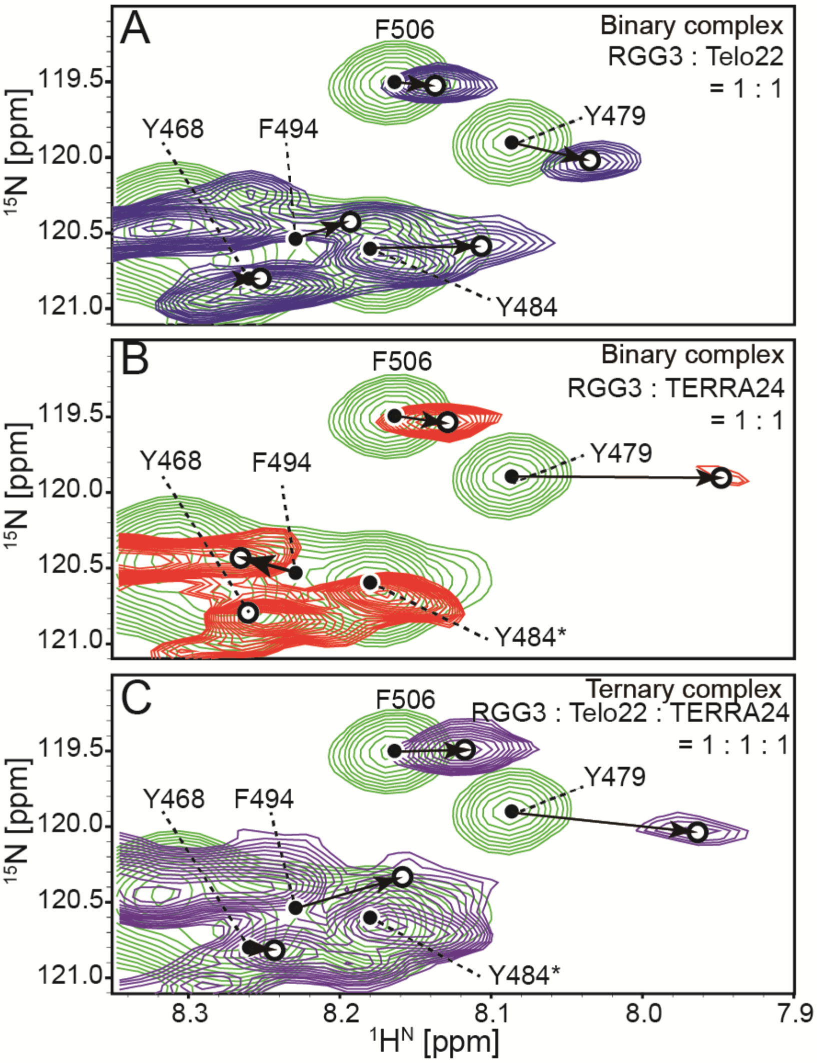

A
Binary complex
RGG3 : Telo22
= 1 : 1
F506
Y479
Y468
F494
Y484
B
Binary complex
RGG3 : TERRA24
= 1 : 1
F506
Y479
Y468
F494
Y484*
C
Ternary complex
RGG3 : Telo22 : TERRA24
= 1 : 1 : 1
F506
Y479
Y468
F494
Y484*
119.5
120.0
120.5
121.0
15N [ppm]
8.3
8.2
8.1
8.0
7.9
1HN [ppm]

Figure S3
